# Self-regulated learning of Natural Sciences and Mathematics future teachers: Learning strategies, self-efficacy, and socio-demographic factors

**DOI:** 10.1186/s41155-021-00203-x

**Published:** 2022-01-04

**Authors:** Ângela Regina dos Reis Arcoverde, Evely Boruchovitch, Natália Moraes Góes, Taylor W. Acee

**Affiliations:** 1 School of Education, State University of Campinas (UNICAMP), Campinas, Brazil; 2 Department of Teacher Education, Science and Letters of the Federal Institute of Education, Science and Technology of Piauí (IFPI), Rua Álvaro Mendes, 94, Centro (Sul), Teresina, PI Brazil; 3 Department of Educational Psychology, School of Education, State University of Campinas (UNICAMP), Avenida Bertrand Russell, 801, Cidade Universitária Zeferino Vaz, Campinas, Brazil; 4 Department of Education, School of Education, State University of Londrina, Highway Celso Garcia Cid S/N, km. 380, Londrina, Paraná Brazil; 5 Department of Curriculum and Instruction, Texas State University, 601 University Drive, San Marcos, TX 78666 USA

**Keywords:** Self-regulation of learning, Self-efficacy to learn, Teacher training, Higher education, Strategic learning, Teaching education programs

## Abstract

Teacher education programs should have as one of their purposes the promotion of self-regulatory skills for learning among students who aspire to be teachers so that they can take a leading role in their learning and foster these skills in their future students. Considering the importance of knowing what students in teacher education programs do to study and learn, as well as how efficacious they feel to deal with academic demands, this study is part of a larger research and aims to investigate the learning and study strategies and self-efficacy for learning beliefs of 220 students enrolled in teacher education programs in Biological Sciences, Chemistry, Physics and Mathematics of a Higher Education Institution in the state of Piauí, and examine them in relation to age, gender, licentiate area, and course semester. Brazilian translations of the Learning and Study Strategies Inventory (LASSI — Third Edition) and the Self-efficacy for Learning Form were used for data collection. Scales were administered in the classrooms both through online platforms and in paper and pencil. Nonparametric inferential statistical approaches were used to test hypotheses regarding group differences. Statistically significant differences were found in LASSI in relation to gender, licentiate area, and course semester. Overall, students in Physics dealt better with anxiety; in Mathematics showed more favorable attitudes towards learning; in Chemistry reported managing their time better; in Biological Science showed significantly lower scores on many scales than did other students. Findings from this study could help inform curricular design decisions regarding teacher education programs and inform the design of interventions to strengthen the learning and study strategies and the self-efficacy for learning beliefs of future teachers.

## Introduction

The tweny-first century is characterized by many changes in the educational system and inevitable transformations in the role of the school and the functions of teachers. Teachers need to create new ways of teaching to better promote students’ learning (Birbal et al., 2018; Coutinho & Miranda, 2019; Guimarães et al., 2021). To face these challenges, training is needed to support teaching practice. Thus, the fundamental role of teacher education programs in promoting and improving the knowledge and skills of future teachers is well acknowledged (Coutinho & Miranda, 2019; Gatti, 2016; Stein & Stein, 2016). One way to promote the improvement of teaching skills in the beginning of teacher education courses is to invest in strengthening the self-regulatory skills of future teachers. Self-regulation of learning is defined as one’s ability to manage cognitive, behavioral, metacognitive, affective, and motivational aspects of oneself to achieve a certain educational goal (Schunk & Zimmerman, 1998; Zimmerman, 2013). Teacher education programs should have as one of its purposes the promotion of skills of future teacher students so that they can control their learning processes and gradually achieve their academic autonomy (Frison et al., 2018; Michalsky & Schechter, 2018; Moos & Ringdal, 2012; Panadero, 2017; Pranke & Frison, 2015; Vosniadou et al., 2021). Strengthening the self-regulatory processes of future teachers when they learn will contribute to the emergence of generations of self-reflective, metacognitive, strategic, and self-regulated teachers (Mansvelder-Longayroux et al., 2007; Vosniadou et al., 2021). Teachers must not only master scientific and specific theoretical knowledge, but they must also acquire methods and strategies that can help their students become agents of their own learning (Coutinho & Miranda, 2019; Gatti, 2016; Pozo & Crespo, 2009).

## Literature review

The literature review addresses theory and research on strategic and self-regulated learning, emphasizing Weinstein’s (1994) model of strategic learning, Zimmerman’s (2000) model of self-regulation, research with Brazilian students and in teacher education programs, and research examining relationships between strategic and self-regulated learning and sociodemographic factors.

### Conceptual models of strategic and self-regulated learning

Though there are several models of self-regulated learning (Panadero, 2017), we chose to focus on both the Model of Strategic Learning (Weinstein, 1994) and the self-regulated learning model (Zimmerman, 2000, 2002) as theoretical frameworks because, as mentioned in Arcoverde et al. (2020), the models of Weinstein (1994) and Zimmerman (2000, 2002) consider learning a complex process, which involves the regulation of cognitive, metacognitive, motivational, affective and behavioral factors.

These two models are congruent yet vary in scope and emphasis (Bortoletto & Boruchovitch, 2013). Zimmerman’s model places greater emphasis on cyclical phases involved in self-regulated learning with strategic planning, usage of learning strategies, and reflection on learning strategies usage occurring during different self-regulatory phases. Weinstein’s model emphasizes self-regulation as one component within a larger strategic learning model consisting of skill, will, self-regulation, and the academic environment. Each model addresses similar factors and makes similar assumptions that students are capable of intentionally improving learning and motivational processes and outcomes. Each model highlights the importance of students’ employment of learning strategies and of having robust self-efficacy beliefs to achieve academic success. Moreover, these models agree that strategic and self-regulated learning can be taught (Ganda & Boruchovitch, 2018; Weinstein, 1994; Zimmerman, 2000, 2002, 2013). Learning and study strategies and self-efficacy for learning are the major variables investigated in the present study.

In the strategic learning model proposed by Weinstein (1994), the components considered controllable by students are divided into three main categories: skill, will, and self-regulation. The skill component refers to knowledge about how to effectively use a variety of learning strategies and thinking skills. The will component consists of motivation and emotions, which can contribute to and detract from academic success. Finally, the self-regulation component refers to factors involved in self-managing learning processes, strategically, on global levels, and in real time. The global level involves the use of a systematic approach, on the part of the student, for long-term learning, for example: managing time in months or years. Self-regulation of real-time learning includes the acts of managing and reducing high anxiety, using metacognition to monitor learning success, monitoring and regulating the use of efficient learning methods and strategies, managing time at a micro level (during a task, for a few hours), focusing attention and maintaining concentration over time, among other possibilities (Weinstein, 1994; Weinstein et al., 2016).

The self-regulated learning model proposed by Zimmerman (2000, 2002, 2013) states that self-regulation consists of an interdependent cyclical process that comprises three phases: forethought, performance or volitional control, and self-reflection. The forethought phase involves setting learning goals and strategically planning to achieve them. The performance or control phase refers to the implementation of actions to achieve the learning goals set and planned in the previous phase. It is at this time that students implement learning strategies, monitor their own performance, and record their progress. In the self-reflection phase, students reflect on the results obtained, considering the goals set in the forethought phase. Reflections in this last phase guide students as to whether they need or not to modify their behavior to achieve success in future learning situations.

### Learning strategies, self-efficacy, and sociodemographic factors

Learning strategies are integrated sequences of procedures that are chosen in order to facilitate the acquisition, storage, and/or use of information or knowledge (McCombs, 2017; Weinstein, 1994; Weinstein & Acee, 2018). By teaching learning and study strategies to students, it is possible to help students become more active in their learning process. According to the literature (McCombs, 2017; Weinstein, 1994; Weinstein & Acee, 2018), students are expected to learn how to use learning and study strategies consciously and effectively, but educational institutions rarely teach them to do so. In fact, students have little access to strategies that can help them learn over the years of schooling (McCombs, 2017; Weinstein & Acee, 2018; Zimmerman & Schunk, 2008). Therefore, there is a need for designing interventions to help students acquire learning strategies to improve these gaps (Bembenutty & Karabenick, 2004; Graham et al., 2005; Zimmerman & Schunk, 2008).

Just as learning and study strategies are relevant for students’ academic performance, beliefs of self-efficacy for learning are also important. Self-efficacy beliefs concern the judgments students make about their ability to perform a given task (Bandura, 1997). The more students consider themselves capable of accomplishing a task, the more they will engage, persist, and strive in its fulfillment. However, if they feel they are not able to accomplish the task successfully, they are less likely to sustain effort towards accomplishing the task (Bandura, 1997; Bzuneck, 2009; Pajares & Olaz, 2008; Schunk & Greene, 2018; Zimmerman, 2000, 2002; Zimmerman & Schunk, 2011).

As mentioned by Arcoverde et al. (2020), Brazilian research on the use of learning and study strategies among Higher Education students is still incipient, if compared to the international literature. This is especially the case regarding teacher education programs in the fields of Natural Sciences and Mathematics (Arcoverde et al., 2020; Ganda & Boruchovitch, 2019).

Although Brazilian studies are still incipient, their findings converge with findings of international studies because both lines of research indicate that university students tend to use learning and study strategies (Boruchovitch et al., 2020; Iqbal et al., 2010; Pavesi & Alliprandini, 2016). They also show some differences in self-reported strategy usage by gender (Bartalo & Guimarães, 2008; Bender & Garner, 2010; Boruchovitch et al., 2020; Braten & Olaussen, 1998; Carpenter et al., 2014; Endo et al., 2017; Lins, 2013; Silva et al., 2016), course semester (Bortoletto, 2011; Boruchovitch et al., 2020; Endo et al., 2017; Marušić et al., 2017; Silva et al., 2016), age (Bortoletto, 2011; Boruchovitch et al., 2020; Braten & Olaussen, 1998; Martins, 2016), and program degree areas (Bartalo & Guimarães, 2008; Martins, 2016). Some authors have identified that male students generally report using more strategies to deal with anxiety than do female students (Bender & Garner, 2010; Boruchovitch et al., 2020; Braten & Olaussen, 1998; Carpenter et al., 2014). Besides gender differences in strategies to deal with anxiety, other studies have, however, pointed out higher reports of learning strategy use among female students in all LASSI scales (Bartalo & Guimarães, 2008), in note taking, in note reviewing, in planning lists, and in environmental structuring (Lins, 2013) and planning strategies (Silva et al., 2016). No differences between genders in the report of the use of learning strategies were found, as well as in other studies (Bortoletto, 2011; Martins, 2016).

Moreover, the literature indicates that first-year students tend to report more strategy use than those who are more advanced in their course of study (Bortoletto & Boruchovitch, 2013; Boruchovitch et al., 2020; Endo et al., 2017). However, Silva et al. (2016) showed that students sampled closer to the end of their college studies mentioned using more learning and study strategies than did their counterparts sampled closer to the beginning of their college studies. Conversely, Ribeiro and Silva (2007) did not find statistically significant differences in students’ self-reported use of learning strategies in relation to course semester.

Results regarding the impact of age in the report of the use of learning strategies have also shown some inconsistencies. While some studies have suggested that older students tend to deal better with anxiety and are more likely to employ cognitive and metacognitive strategies and seek academic support when compared to their younger counterparts (Bortoletto & Boruchovitch, 2013; Boruchovitch et al., 2020; Braten & Olaussen, 1998), other studies have found the relationship between age and learning and study strategies to be null (Bartalo & Guimarães, 2008; Silva et al., 2016), and one study indicated that younger students mentioned using more learning strategies than older students (Martins, 2016). Concerning students’ undergraduate program degree areas, studies have shown no statistically significant differences in self-reported learning and study strategies among students from different degree programs such as Librarianship and Archival Science (Bartalo & Guimarães, 2008), Pedagogy, Physical Education, Biological Sciences, Letters and Literature, Mathematics and Chemistry (Boruchovitch et al., 2020) and Health, Humanities and Exact Science areas (Lins, 2013).

Sociodemographic differences in university students’ self-efficacy for learning beliefs were also found in the literature. Ozan et al. (2012) pointed out that female students showed higher self-efficacy for learning beliefs than did males. However, other studies have indicated that male students tend to perceive themselves as having more self-efficacy for learning when compared to females (Altunsoy et al., 2010; Teixeira & Costa, 2018). In addition, there were also studies which have not found gender differences in self-efficacy for learning beliefs (Jakešováa et al., 2015; Martins, 2016). Some studies had also identified that younger students were more likely to have higher self-efficacy for learning beliefs than did their older counterparts (Erb & Drysdale, 2017; Martins, 2016). The study conducted by Teixeira and Costa (2018) found that older students tend to consider themselves more self-efficacious than those who are younger. Jakešováa et al. (2015) observed no age-related differences in students’ beliefs of self-efficacy for learning. Altunsoy et al. (2010) concluded that students who were more advanced in their courses reported being more self-efficacious for learning. Additionally, regarding program degree areas, Ozan et al. (2012) found that students of School of Education reported higher levels of self-efficacy for learning, when compared to those of Faculty of Agriculture. Martins (2016), in his study, comparing the courses of Psychology, Production Engineering, Physical Education and Veterinary Medicine found no significant program degree area related differences in students’ self-efficacy for learning beliefs.

The importance of identifying what Brazilian students in teacher education programs do to study and learn, how efficacious they feel to deal with academic demands, as well as the relevance of understanding how sociodemographic variables impact students’ report of the use of learning and study strategies and their self-efficacy beliefs for learning, coupled with the scarcity of research on these variables in Brazilian samples motivated the present study. In consonance, the objective of this study was to investigate the learning and study strategies and the self-efficacy for learning beliefs of licentiate students of all the teacher education programs (Biological Sciences, Chemistry, Physics, and Mathematics) offered in a Brazilian Federal Education Institution in Piauí-Brazil and examine them in relation to age, gender, licentiate area, and course semester. We believe that the present research is relevant to educators working in teacher education programs and scholars studying strategic and self-regulated learning and motivation in college students. We hope that the results of this study may be useful not only to rethink the education offered in teacher education programs but also to guide the design of interventions aimed at strengthening learning and study strategies and self-efficacy for learning beliefs of future teachers.

### Research questions

To what extent do undergraduate students majoring in Biological Sciences, Chemistry, Physics, and Mathematics report using learning and study strategies and holding robust beliefs of self-efficacy for learning?

Do students’ learning and study strategies scores as well as their self-efficacy for learning beliefs scores differ by age, gender, licentiate area, and course semester?

### Hypotheses

H1: Students will report low scores in learning and study strategies and will have moderate beliefs of self-efficacy for learning.

H2: The reports of the use of learning and study strategies and of self-efficacy for learning beliefs will differ by age, gender, licentiate area, and course semester.

Considering that this study is exploratory in nature and that the literature is scarce and inconclusive regarding the direction of these differences, we do not have specific hypotheses about how such differences will emerge in the sample.

## Method

This study is exploratory and had a cross-sectional, descriptive, and correlational design.

### Participants

The sample consisted of 220 students from sixteen (100%) classes of licentiate degree programs in areas of Biological Sciences (*n* = 70; 31.82%), Chemistry (*n* = 51; 23.18%), Physics (*n* = 53; 24.09%), and Mathematics (*n* = 46; 20.91%) of a Brazilian Federal Education Institution in the state of Piauí. The age of the participants ranged from 17 to 56 years and the mean age was 22.34 years (*SD*=6.485). Of the total sample, 93 (42.27%) were under 20 years old, 100 (45.45%) between 20 and 29 years old, and 25 (11.36%) over 30 years old and 2 (0.90%) did not respond to the age question. As for gender, 101 (45.91%) were female and 119 (54.09%) were male. Regarding course semester, 131 students (59.54%) were from the first and second semesters, 48 (21.82%) were from the third to the fifth semesters, and 41 (18.64%) from the sixth to the eighth. Students self-reported being white (*n* = 22, 18.97%), black (*n* = 14, 12.07%), brown (*n* = 78, 67.24%) and others (*n* = 2, 1.72%). Most of the students reported having attended high school in public schools (*n* = 81; 69.83%), 27 completed it in private schools (23.28%) and 8 attended part in public schools and part in private schools (6.90%).

### Data collection instruments

#### Sociodemographic questionnaire

The sociodemographic questionnaire contained four multiple-choice questions about students’ age, gender, licentiate area, and course semester which were used to describe the participants.

#### Learning and Study Strategies Inventory Third Edition (LASSI 3rd ed.; Weinstein et al., 2016) — Translated and adapted by Boruchovitch et al. (2019)

Boruchovitch et al. (2019) had described in detail the process of translating the LASSI (3rd ed.) into Portuguese and adapting it for use with university students in Brazil. In short, the process involved an initial translation conducted by three Brazilian researchers well-versed in research on the LASSI and the Model of Strategic Learning and fluent in both Brazilian Portuguese and American English. Back translation carried out by an expert translator with a Ph.D. in English and fluent in Portuguese led to further refinements and those refinements were again back translated. After those revisions were complete, the translated version was submitted to two expert judges from Brazil and the back translation was sent to one of the original authors of the LASSI, all of whom confirmed the adequacy of the translation and adaptation with no suggested revisions.

The LASSI (3rd ed.) is a Likert-type scale containing 60 items, with 5 choices for answers: not at all typical of me, not very typical of me, somewhat typical of me, fairly typical of me, and very much typical of me. The 3rd edition (Weinstein et al., 2016) was developed with 3 main purposes: (a) to refine the wording of some items from the previous editions of 1988 and 2002; (b) to include a new scale — Using Academic Resources — replacing the Study Aids scale, to better reflect the advances of contemporary educational psychology and postsecondary educational practice; and (c) to decrease its application time by reducing it from 80 to 60 items.

Of its current 60 items, 34 have reverse scores, due to the directionality in which they were written. The LASSI items are subdivided into 10 scales: Anxiety, Attitude, Concentration, Information Processing, Motivation, Selecting Main Ideas, Self Testing, Test Strategies, Time Management, and Using Academic Resources. Each scale consists of 6 items. Weinstein et al. (2016) mentioned that LASSI scores can be analyzed considering each scale separately. Thus, scores can range from 6 (minimum score) to 30 (maximum score) in each scale. The 10 scales are associated with either the Skill, Will, or Self-Regulation components of strategic learning according to the Model of Strategic Learning (Weinstein et al., 2016). More precisely, Information Processing, Selecting Main Ideas, and Test Strategies scales are related to the Skill component. Anxiety, Attitude, and Motivation scales are associated with the Will component. Concentration, self testing, time management, and using academic resources are related to the self-regulation component.

The Anxiety scale assesses the degree to which students worry about college and their academic performance (example item: “when I am taking a test, worrying about doing poorly interferes with my concentration”). The Attitude scale examines students’ attitudes and interests regarding college and reaching academic success (example item: “I have a positive attitude about attending my classes”). The Concentration scale assesses students’ ability to direct and maintain their attention on academic tasks (example item: “I find it difficult to maintain my concentration while doing my coursework”). The Information Processing scale examines the extent to which students use visual and verbal elaboration, organizational, and other active-thinking strategies to help them learn and remember new information (example item: “to help me remember new principles we are learning in class, I practice applying them”). The Motivation scale assesses students’ diligence, self-discipline, and effort to accomplish their academic tasks (example item: “When work is difficult, I either give up or study only the easy parts”).

The Selecting Main Ideas scale assesses students’ skills at tracing important information to study in various learning situations in college (example item: “I have difficulty identifying the important points in my reading”). The Self Testing scale measures students’ use of strategies for monitoring their comprehension of course material and checking their ability to demonstrate their learning (example item: “I stop periodically while reading and mentally go over or review what was said”). The Test Strategies scale concerns the strategies used by students both at the time of preparation for a test and at the time the test is taken (example item: “I have difficulty adapting my studying to different types of courses”). The Time Management scale measures the use of time management principles and practices by students when performing academic tasks (example item: “when I decide to study, I set aside a specific length of time and stick to it”). Lastly, the Using the Academic Resources scale assesses the students’ willingness to use different academic resources (example item: “when I am struggling in one or more courses, I am too embarrassed to admit it to anyone”). All 10 scales have high internal consistencies, measured by Cronbach’s Alpha, in studies carried out in large samples of American students. The values ranged from 0.76 to 0.87.

In Brazil, the internal consistency of the total LASSI and each of its scales was estimated in a study conducted by Boruchovitch et al., 2020 in a sample of 163 students enrolled in a teacher education program at a public university in the state of São Paulo. The alpha of the Cronbach value of the total LASSI was 0.911. With the exception of the scale of using academic resources (*α* =0.386), the reliability of the LASSI scales was high, ranging from *α* = 0.717 to *α* = 0.843 in nine of the ten scales (Pestana & Gageiro, 2014).

The alpha of the Cronbach values was also estimated for the present sample. The reliability of LASSI ranged from *α* = 0.606 to *α* = 0.899, with the exception of using the Academic Resources scale which was also low (*α* = 0.448). The alpha values of the scales of attitude, motivation, and test strategies ranged from 0.606 to 0.674. These values are somehow lower than in the previous Brazilian study, but can be considered acceptable in the Humanities (Pestana & Gageiro, 2014; Prieto & Muñiz, 2000).

With the exception of using the Academic Resources scale, the reliabilities obtained in the Brazilian study were similar to those found in American studies (Weinstein et al., 2016). In fact, the Cronbach alpha value for using the Academic Resources scale could have been improved with the withdrawal of some of its items. However, for now, the decision was not to exclude any item from this scale, since the psychometric properties of the Brazilian version of LASSI are still under study in a much larger sample.

#### Self-Efficacy for Learning Form (Zimmerman & Kitsantas, 2005) — Translated to Portuguese by Boruchovitch and Ganda (2010)

This Likert-type scale consisted of 19 items that refer to the self-efficacy beliefs related to 3 academic activities: study, preparation for tests, and note-taking in class. The options assumed values ranging from 0 to 100%, according to the following gradation: 0% (Definitely cannot do it), 30% (Probably cannot do it), 50% (Maybe can do it), 70% (Probably can do it), and 100% (Definitely can do it). The total score ranged from 0 to 100, calculated by taking the mean of all 19 items (Simmons & Lehmann, 2015). Higher scores reflect more positive beliefs in self-efficacy for learning. Some examples of items are as follows: “When you are trying to understand a new topic, can you associate new concepts with the old ones sufficiently well to remember them?” and “When you think you did poorly in a test you just finished, can you go back to your notes and locate all the information you had forgotten?”

The questionnaire was translated into Portuguese by Boruchovitch and Ganda (2010) after obtaining formal consent from the authors. To ensure accuracy, the form was independently translated by two fluent English speakers. The translations were then compared and discussed to determine the final Brazilian version. Back translation procedures were also employed.

The internal consistency of the scale, measured by Cronbach’s alpha, was 0.97 in a study conducted with 223 undergraduate students (Zimmerman & Kitsantas, 2007). In a Brazilian study carried out with a sample of 884 undergraduate students (Boruchovitch et al., 2015), the alpha value was high (*α* = 0.99) and was (*α* = 0.910) in another study based on a sample of 109 university students (Ganda & Boruchovitch, 2018). The reliability of the self-efficacy for learning beliefs form was also estimated for the present sample. It was also high (*α* = 0.910) and similar to that obtained by the original authors (Zimmerman & Kitsantas, 2007) and to the previous Brazilian studies (Boruchovitch et al., 2015; Boruchovitch et al., 2019). Temporal stability was also measured (Balsas & Boruchovitch, 2015). A high and statistically significant correlation was found between the two applications (*α* = 0.89; *p* < 0.001).

### Data collection procedures

The project was submitted and approved by the Research Ethics Committee of the School of Education of a public university, in accordance with the current regulations of the National Health Council, resolution n. 506/16, which establishes the ethical issues of research conducted with human beings in Brazil (CAAE protocol: 02209218.6.0000.8142). Then, an invitation letter was sent to the undergraduate chairs requesting authorization to carry out the research. They showed great interest in the research due to its relevance to understanding and improving university student learning.

Data collection was scheduled after consulting the most appropriate days and times, according to the teachers of each licentiate area. Data were collected in all of the sixteen classes available at the institution. Students in those classes were pursuing licentiate degrees in Biological Sciences, Chemistry, Physics, or Mathematics. The application of the instruments took place in the classroom and, in all classes, the same procedures were adopted. Before starting the data collection, the researcher explained to the students the purpose of the research, how the collection would occur, and its confidential nature. Soon after, the researcher made the research link available to participants. Students were asked to click the link and register their emails. The researcher then sent another link to students’ registered emails with an invitation for participation in the research. By accessing the link using smartphones, laptops, and/or tablets, students were directed to the *Autorregular* Platform, which hosted the informal consent form, sociodemographic questions, LASSI (web version), and Self-efficacy for Learning Form. Students who were unable to access the link due to Internet connectivity problems answered all the instruments in paper-and-pencil format (*n* = 102; 46.36%). Data collection in each class lasted approximately 50 min.

### Data analysis procedures

The statistical Package for the Social Science – SPSS version 22 was used to analyze the data. Descriptive and comparative analyses were carried out. After verifying that the data did not present normal distribution by the values obtained in the Shapiro-Wilk and Kolmogorov-Smirnov tests, the Mann-Whitney test was used to compare the variables between two groups and the Kruskal-Wallis test to compare the variables between three groups. Cronbach’s alpha was used to analyze the internal consistency of the scales. Alpha values above 0.70 indicate high internal consistency (Shavelson, 2009). The level of significance adopted in the present study for statistical tests was 5% (*p* < 0.05).

## Results

Table 1 presents the results of the alpha values and descriptive analysis of the Brazilian translation of the LASSI 3rd Ed. and the Brazilian translation of self-efficacy for learning beliefs form.

**Table 1:** Descriptive analysis of the sample in LASSI 3^rd^ Ed. and self-efficacy for learning beliefs form

Scales	*α*	Mean	Sd	Min	Median	Max
Learning and Study Strategies Inventory – LASSI						
*N* = 220						
Anxiety	0.821	2.88	0.92	1.00	3.00	4.83
Attitude	0.606	4.13	0.53	2.33	4.17	5.00
Concentration	0.739	3.44	0.74	1.17	3.50	4.83
Information processing	0.723	3.64	0.68	1.33	3.67	5.00
Motivation	0.621	3.71	0.63	1.67	3.83	5.00
Selection of the main ideas	0.714	3.45	0.72	1.17	3.50	4.83
Self-testing	0.726	3.13	0.81	1.00	3.17	5.00
Test strategies	0.674	3.52	0.70	1.83	3.50	5.00
Time management	0.704	3.14	0.73	1.33	3.17	4.83
Using academic resources	0.448	3.05	0.63	1.00	3.00	4.67
Total LASSI	0.899	3.41	0.43	2.20	3.44	4.57
Self-efficacy for Learning Form						
*N* = 220						
Total self-efficacy	0.910	66.5	13.62	12.1	69.0	99.0

Both instruments were translated to Brazilian Portuguese

The LASSI total mean and median (*M* = 3.41; Mdn = 3.44) suggest that students generally use learning and study strategies. The attitude scale had the highest scores (*M* = 4.13; Mdn = 4.17). The anxiety scale had the lowest mean and median (*M* = 2.88; Mdn = 3.00), followed by the use of the Academic Resources scale (*M* = 3.05; Mdn = 3.00), self-testing (*M* = 3.13; Mdn = 3.17) and time management (*M* = 3.14; Mdn = 3.17). The standard deviation of the total LASSI was 0.43 and of the scales varied from 0.53 to 0.92. Overall, there were no large variations in students’ responses on the different LASSI scales. The scores obtained (*M* = 66.5; Mdn = 69.0) on the self-efficacy for learning beliefs form indicate that students have moderate self-efficacy for learning beliefs. The standard deviation was 13.62. Table 2 shows the comparison of the Brazilian translation of the LASSI 3rd Ed. and the Brazilian translation of self-efficacy for learning beliefs form by gender and age.

**Table 2:** Scores in LASSI 3rd Ed. and in Self-efficacy for Learning Form by gender and age

Learning and Study Strategies Inventory — LASSI											
Gender											
	Female (*n* = 109)				Male (*n* = 119)				*Z*	*p*	
Scale	M	Sd	Mdn		M	Sd	Mdn				
ANX	2.53	0.90	2.50		3.18	0.84	3.33		5.11	**< 0.001**	
ATT	4.12	0.56	4.17		4.14	0.50	4.17		0.28	0.780	
CON	3.42	0.74	3.50		3.46	0.73	3.50		0.21	0.834	
INP	3.63	0.62	3.67		3.65	0.74	3.67		0.29	0.773	
MOT	3.69	0.61	3.83		3.72	0.64	3.83		0.34	0.737	
SMI	3.54	0.72	3.50		3.37	0.71	3.50		1.71	0.086	
SFT	3.16	0.77	3.17		3.11	0.84	3.17		0.25	0.803	
TST	3.52	0.72	3.50		3.53	0.68	3.50		0.23	0.818	
TMT	3.14	0.68	3.17		3.14	0.77	3.17		0.12	0.905	
UAR	3.07	0.67	3.17		3.03	0.59	3.00		0.50	0.615	
Total	3.38	0.44	3.43		3.43	0.42	3.45		0.54	0.589	
Self-Efficacy for Learning Form											
Gender											
	Female (*n* = 109)				Male (*n* = 119)				*Z*	*p*	
Scale	M	Sd	Mdn		M	Sd	Mdn				
Total AE	64.73	14.26	66.84		67.96	12.93	69.47		1.52	0.127	
Learning and Study Strategies Inventory — LASSI											
Age											
	< 20 years (*n* = 93)			20–29 years (*n* = 100)			≥ 30 years (*n* = 25)			*χ*^2^	*p*
Scale	*M*	Sd	Mdn	*M*	Sd	Mdn	*M*	Sd	Mdn		
ANX	2.89	0.91	3.00	2.85	0.97	2.92	3.01	0.87	2.83	0.38	0.825
ATT	4.16	0.52	4.17	4.12	0.54	4.17	4.07	0.53	4.00	0.90	0.637
CON	3.54	0.69	3.67	3.35	0.77	3.33	3.43	0.75	3.67	2.83	0.242
INP	3.72	0.59	3.67	3.59	0.78	3.67	3.55	0.60	3.50	1.89	0.389
MOT	3.75	0.60	3.83	3.70	0.68	3.67	3.59	0.44	3.67	2.57	0.277
SMI	3.47	0.72	3.50	3.48	0.73	3.50	3.23	0.69	3.17	2.99	0.224
SFT	3.17	0.76	3.17	3.08	0.83	3.08	3.14	0.90	3.17	0.62	0.735
TST	3.63	0.68	3.67	3.44	0.72	3.42	3.45	0.63	3.33	3.78	0.151
TMT	3.18	0.70	3.33	3.09	0.75	3.17	3.19	0.81	3.33	0.93	0.629
UAR	3.12	0.64	3.17	2.96	0.62	3.00	3.13	0.65	3.17	3.87	0.144
Total	3.46	0.41	3.53	3.36	0.46	3.38	3.38	0.39	3.37	3.57	0.168
Self-efficacy for Learning Form											
Age											
	< 20 years (*n* = 93)			2–29 years (*n* = 100)			≥ 30 years (*n* = 25)			*χ*^2^	*p*
Scale	*M*	Sd	Mdn	*M*	Sd	Mdn	*M*	Sd	Mdn		
Total AE	67.8	12.5	68.9	65.0	14.4	68.2	66.2	13.6	66.3	1.20	0.549

Legend: *M* Mean, *Mdn* Median, *AE* self-efficacy, *ANX* Anxiety, *ATT* Attitudes, *CON* Concentration, *INP* Information processing, *MOT* Motivation, *SMI* Selection of main ideas, *SFT* Self-testing, *TST* Test strategies, *TMT* Time management, *UAR* Using academic resources

Data in Table 2 show that significant differences were found in LASSI scores in relation to gender. Male students (*M* = 3.18; Mdn = 3.33; *p*<0.001) had significantly higher scores on the Anxiety scale when compared to females. Furthermore, there were no statistically significant differences in the age and Self-Efficacy for Learning Form in relation to any of the variables investigated.

Table 3 shows the comparison of the Brazilian translation of the LASSI 3rd Ed. and the Brazilian translation of self-efficacy for learning beliefs form by licentiate area and semester of the course.

**Table 3:** Scores in LASSI 3rd Ed. and in self-efficacy for learning by licentiate area and semester

Learning and Study Strategies Inventory — LASSI														
Licentiate area														
	Biological Sciences (*n* = 70)			Physics (*n* = 53)			Mathematics (*n* = 46)			Chemistry (*n* = 51)			*χ*^2^	*p*
Scale	M	Sd	Mdn	M	Sd	Mdn	M	Sd	Mdn	M	Sd	Mdn		
ANX	2.78	0.97	2.83	3.27	0.89	3.33	2.62	0.92	2.58	2.87	0.79	2.83	14.31	0.003
ATT	4.03	0.59	4.08	4.08	0.58	4.17	4.33	0.42	4.33	4.14	0.42	4.17	8.23	0.041
CON	3.39	0.72	3.50	3.50	0.71	3.67	3.32	0.86	3.33	3.57	0.66	3.67	2.15	0.542
INP	3.75	0.65	3.67	3.60	0.73	3.67	3.66	0.73	3.67	3.50	0.62	3.50	5.22	0.156
MOT	3.73	0.56	3.83	3.64	0.65	3.67	3.77	0.62	3.75	3.69	0.69	3.83	1.13	0.770
SMI	3.45	0.75	3.50	3.35	0.69	3.33	3.46	0.76	3.50	3.53	0.68	3.67	2.20	0.531
SFT	3.21	0.79	3.17	3.15	0.88	3.17	2.95	0.75	3.00	3.16	0.80	3.17	2.42	0.489
TST	3.46	0.68	3.50	3.51	0.63	3.50	3.58	0.73	3.67	3.58	0.76	3.50	1.22	0.748
TMT	3.00	0.63	3.00	3.17	0.83	3.17	3.02	0.76	3.08	3.41	0.65	3.33	10.76	0.013
UAR	3.00	0.68	3.00	3.03	0.52	3.00	3.06	0.63	3.00	3.14	0.67	3.17	0.93	0.819
Total	3.38	0.43	3.45	3.43	0.44	3.45	3.38	0.42	3.36	3.46	0.44	3.48	1.02	0.797
Self-efficacy for Learning Form														
Licentiate area														
	Biological Sciences (*n* = 70)			Physics (*n* = 53)			Mathematics (*n* = 46)			Chemistry (*n* = 51)			*χ*^2^	*p*
Scale	*M*	Sd	Mdn	*M*	Sd	Mdn	*M*	Sd	Mdn	*M*	Sd	Mdn		
Total AE	66.1	12.0	66.8	66.7	13.4	69.5	67.9	13.9	69.5	65.4	15.8	68.9	0.60	0.896
Learning and Study Strategies Inventory — LASSI														
Semester of the course														
	Semesters 1 and 2 (*n* = 131)				Semesters 3–5 (*n* = 48)			Semesters 6–8 (*n* = 41)				*χ*^2^	*p*	
Scale	*M*	Sd	Mdn		*M*	Sd	Mdn	*M*		Sd	Mdn			
ANX	2.97	0.88	3.00		2.77	0.99	2.83	2.74		0.96	3.00	2.28	0.320	
ATT	4.19	0.49	4.17		4.14	0.54	4.17	3.93		0.60	4.00	6.13	0.047	
CON	3.53	0.68	3.67		3.49	0.67	3.67	3.11		0.89	3.17	8.58	0.014	
INP	3.65	0.64	3.67		3.61	0.66	3.58	3.63		0.85	3.67	0.32	0.853	
MOT	3.79	0.56	3.83		3.61	0.65	3.67	3.57		0.75	3.67	3.81	0.149	
SMI	3.48	0.71	3.67		3.45	0.70	3.50	3.35		0.78	3.33	1.31	0.521	
SFT	3.27	0.81	3.33		2.97	0.68	3.00	2.87		0.84	2.83	12.49	0.002	
TST	3.61	0.67	3.67		3.48	0.67	3.50	3.30		0.76	3.17	6.49	0.039	
TMT	3.22	0.72	3.33		3.13	0.69	3.17	2.91		0.75	2.83	5.40	0.067	
UAR	3.15	0.63	3.17		2.94	0.58	3.00	2.87		0.66	3.00	7.10	0.029	
Total	3.49	0.41	3.55		3.36	0.38	3.40	3.23		0.50	3.18	11.47	0.003	
Self-efficacy for Learning Form														
Semester of the course														
	Semesters 1 and 2 (*n* = 131)				Semesters 3–5 (*n* = 48)				Semesters 6–8 (*n* = 41)			*χ*^2^	*p*	
Scale	*M*	Sd	Mdn		*M*	Sd	Mdn		*M*	Sd	Mdn			
Total AE	68.4	12.3	69.5		62.1	15.9	66.0		65.2	13.8	67.4	5.42	0.067	

Legend: *M* Mean, *Mdn* Median, *AE* self-efficacy, *ANX* Anxiety, *ATT* Attitudes, *CON* Concentration, *INP* Information processing, *MOT* Motivation, *SMI* Selection of main ideas, *SFT* Self-testing, *TST* Test strategies, *TMT* Time management, *UAR* Using academic resources

Data in Table 3 show that significant differences were found in LASSI scores in relation to licentiate area and course semester. Physics students (*M* = 3.27; Mdn = 3.33; p = 0.003) had significantly higher scores on the Anxiety scale when compared to students from other licentiate areas. Students who were pursuing licentiate degrees in Mathematics and Chemistry obtained significantly higher scores on the attitude (*M* = 4.33; Mdn = 4.33; *p* = 0.041) and time management (*M* = 3.41; Mdn = 3.33; *p* = 0.013) scales, when compared to those in Biological Sciences.

Students in the beginning of their course of study (i.e., first and second semesters) showed statistically significantly higher scores on the attitude (*M* = 4.19, *p* = 0.047), self-testing (*M* = 3.27, *p* = 0.002), test taking strategies (*M* = 3.61, *p* = 0.039), and use of academic resources scales (*M* = 3.15, *p* = 0.029) and LASSI total (*M* = 3.49, *p* = 0.003) when compared to those who were in the end of their course of study (i.e., six to eight semesters) whose scores in those scales were respectively for attitude (*M* = 3.93), self-testing (*M* = 2.87), test taking strategies (*M* = 3.30); use of academic resource (*M* = 2.87) and LASSI total (*M* = 2.87). Moreover, beginners reported significantly higher scores on the concentration scale (*M* = 3.53, *p* = 0.014) than did both students who were in the middle (i.e., third to fifth semesters *M* = 3.49) and in the end (i.e., sixth to eight semesters *M* = 3.11) of their course of study. Furthermore, there were no statistically significant differences in Self-Efficacy for Learning Form scores in relation to any of the variables investigated.

## Discussion

This study aimed to investigate the learning and study strategies and self-efficacy for learning beliefs of students pursuing a licentiate degree in Biological Sciences, Chemistry, Physics, or Mathematics, and examine them in relation to age, gender, licentiate area, and course semester. The findings from this study help to expand research on strategic and self-regulated learning to Brazilian higher education. More specifically, the results help to show that theoretical constructs described in Weinstein’s (1994) Model of Strategic Learning and measured through the ten scales of the Learning and Study Strategies Inventory (Weinstein et al., 2016) are measurable for and applicable to Brazilian students pursuing a licentiate degree. Results show that students reported moderate usage of learning and study strategies and thus have room to improve in these areas. In addition, the present research shows that students reported usage of learning and study strategies varies by gender, licentiate area, and course semester. Findings about gender differences in learning strategies related to anxiety aligned with previous research (e.g., Bender & Garner, 2010). Findings regarding licentiate area and course semester were novel, as these factors have not been the focus of much previous research on strategic learning. Accordingly, findings from this study open new questions as to the role of contextual and temporal factors in shaping learning and study strategies during college, an area under explored within the Model of Strategic Learning.

The study also examined self-efficacy for learning, which is an important aspect of self-regulated learning (Zimmerman, 2000; Zimmerman & Kitsantas, 2007). Like the findings for learning and study strategies, students’ self-efficacy for learning scores were moderate and showed room for improvement. Unlike the findings for learning and study strategies, no differences by gender, licentiate area, course semester, or age were observed. Together these findings suggest that learning and study strategies and self-efficacy for learning may vary in their sensitivity to the influences that underpin the observed differences in gender, licentiate area, and course semester. What follows is a more detailed discussion of these findings and related research organized by each hypothesis.

The first hypothesis was that students would report low scores in learning and study strategies and will have moderate beliefs of self-efficacy for learning. This hypothesis was partially confirmed since students scored higher in some scales and lower in others.

Results show that students generally report using learning and study strategies, with some strategies being reported more than others. These findings converge with research on strategic and self-regulated learning (Arcoverde et al., 2020; Iqbal et al., 2010; Pavesi & Alliprandini, 2016). The attitude scale showed the highest score. As this scale examines the value that students attach to the educational institution and the importance to achieve educational goals successfully, results indicated that students seem to value their course and academic performance as a way to achieve future professional success. This is a positive result and is aligned with those found by Bartalo and Guimarães (2008), Boruchovitch et al. (2020) and Endo et al. (2017) but differs from the results of Iqbal et al. (2010) which pointed out that Pakistani university students did not demonstrate good attitudes towards university.

The lowest score in LASSI, on the other hand, was on the Anxiety scale. This result suggests that the students who composed the sample do not cope well with anxiety. They feel fear and worry about possible failures. This, in turn, can prevent them from concentrating and focusing attention on thoughts and behaviors relevant to the successful completion of academic tasks. This result diverges from those of Boruchovitch et al. (2020) and Endo et al. (2017) whom both found students’ lowest scores were on the self-testing scale. They also differ from those of Bartalo and Guimarães (2008) in which the lowest scores emerged in the time management scale and those of Ganda and Boruchovitch (2018) in which lowest scores occurred on the motivation scale and of Iqbal et al. (2010) in which lowest scores were in attitude scale.

Furthermore, hypothesis 1 was confirmed regarding self-efficacy for learning beliefs. The students of the present study seem to have moderate beliefs of self-efficacy for learning. This result converges with that of Altunsoy et al. (2010) but differs from those found by Erb and Drysdale (2017) and Jakešováa et al. (2015). Such authors found high beliefs of self-efficacy for learning among university students. Having good attitudes towards learning and adequate beliefs about one's own ability to perform academic tasks successfully are factors that positively influence academic performance and future professional life (Bandura, 1997; Pajares & Valiante, 2006).

When comparing the results of LASSI in relation to age, gender, licentiate area, and course semester, statistically significant differences emerged in relation to gender, licentiate area, and semester of the course as expected. Hypothesis 2 was partially confirmed, except for age and for self-efficacy beliefs for learning as it will be discussed in detail next.

The only gender difference found was for male students, who reported handling academic anxiety more effectively than female students. This result converged with the study by Bender and Garner (2010), Boruchovitch et al. (2020), Braten and Olaussen (1998) and Carpenter et al. (2014)) but diverged from studies conducted by Endo et al. (2017) who found no differences between genders except that information processing was higher for male students. Findings also diverge from the research of Bartalo and Guimarães (2008) which found that female students had higher scores in all LASSI scales. As gender is an important variable associated with discrepancies in results, further research is recommended to deepen our understanding of its impact on variables associated with learning.

Students in the initial semesters of their course of study exceeded those in their final semesters in attitude, self-testing, test taking, using academic resources, concentration and in the total LASSI scales. Also, for the concentration scale, students who were in the middle of their course of study had significantly higher scores than those in their initial semesters. Studies such as those of Boruchovitch et al. (2020), Bortoletto (2011) and Endo et al. (2017) also identified higher levels of learning and study strategies in students in the initial semesters. However, results from research carried out by Marušić et al. (2017), Ribeiro and Silva (2007) and Silva et al. (2016) were contrary to those of the present study, revealing that students of more advanced semesters reported using more learning and study strategies than did those who were in the initial semesters. The present study also converges with research that has shown attitudinal decline of STEM students in their first two years of college (Robinson et al., 2019). Over two years, students reported declines in interest, utility value, attainment value, and perceived competence in their course of study, and higher perceived costs regarding effort and negative emotions. Relating the time in one’s course of study to the development of expertise, Alexander (2004) suggests potential qualitative and quantitative differences in learning, motivation, and strategic processing when progressing from novice to expert.

Moreover, the academic domain of one’s course of study is also suggested to influence learning, motivation, and strategic processing (Alexander, 2004). Converging with this general notion, findings from the present study suggest some differences across licentiate areas in their self-reported use of learning and study strategies. Students in Physics licentiate program dealt better with anxiety. Those who were in Mathematics licentiate program showed more favorable attitudes towards academic success and those who were in Chemistry licentiate program reported managing their time better. Students who were in Biological Science licentiate program showed significantly lower scores on anxiety, attitudes and time management scales when compared to those in other licentiate areas. It is unclear why these differences between licentiate areas were found in the present study, as research comparing students learning and study strategies by degree program areas is limited, and the few research studies we found suggested no differences in learning strategies by degree program areas (Bartalo & Guimarães, 2008; Boruchovitch et al., 2020; Lins, 2013). Most studies examining differences in strategic learning and motivation by academic domain have examined variance within students across different academic domains (e.g., Bong, 2003), and results suggest that students tend to develop subject-matter specific approaches, motivational beliefs, and attitudes. The present study examines variation between groups of students based on their chosen licentiate area within teacher education programs. Findings from the present study revealed statistically significant differences in the reported use of learning and study strategies in relation to licentiate area, showing that further research is needed to determine factors that may explain these differences. Students in different licentiate areas within teacher education programs have different required coursework and program communities of faculty, teachers’ different approaches to teaching and to students’ learning, staff, and students with whom they interact, all of which could differentially shape students’ use of learning and study strategies and self-efficacy. Moreover, one can speculate that such differences might have emerged as a function of specific characteristics of this particular sample.

This finding that students who were in biological science licentiate program reported lower learning and study strategies than students from other licentiate areas has practical implications for the local institution and teachers of this licentiate degree program, because it emphasizes a need to facilitate learning and study strategies development for students in biological sciences licentiate program, especially regarding anxiety control, attitudes to study, and time management, given that these areas were lower for students in biological sciences. Approaches for teaching learning strategies have been widely discussed in the literature (Weinstein, 1994; Weinstein & Acee, 2018) such as teaching students about learning theories and the self-regulated application of learning strategies (Zimmerman, 2013) and using modeling with guided practice and feedback to scaffold students’ strategy knowledge and metacognitive awareness and autonomous usage of learning strategies. By including activities that favor self-reflection and self-assessment in their classroom practices, teachers can help these students develop greater awareness about their behaviors and attitudes towards learning.

As in the present study, in Bartalo and Guimarães (2008) as well as in Ribeiro and Silva (2007) research, no significant differences emerged when examining the results of LASSI in relation to age. This result differed from those obtained by Carpenter et al. (2014)) and Braten and Olaussen (1998) who found that older students significantly outperformed younger students on LASSI attitude scale.

No statistically significant differences were found in students’ self-efficacy for learning beliefs and age, gender, licentiate area and semester of the course. Results from studies conducted by Martins (2016) converged with those of the present study as no statistically significant differences in self-efficacy beliefs were found in relation to gender and licentiate area. Research undertaken by Jakešováa et al. (2015) also did not identify significant differences in self-efficacy beliefs in relation to gender and age. However, the results of the present study diverged from the literature, when significant differences in self-efficacy for learning beliefs were identified in relation to gender (Altunsoy et al., 2010; Ozan et al., 2012; Teixeira & Costa, 2018), age (Erb & Drysdale, 2017; Martins, 2016; Teixeira & Costa, 2018), semester (Altunsoy et al., 2010) and licentiate area (Ozan et al., 2012). Whereas research shows differences in self-efficacy within students across different academic domains (Bong, 2003), no differences between students of different licentiate areas could be explained because students tend to choose to pursue degree programs in academic domains in which they are reasonably self-efficacious, essentially limiting the lower range of self-efficacy scores.

Our society demands individuals who are critical, strategic, self-reflective, and proactive when it comes to learning itself. Learning autonomously requires students to have strategic learning techniques that involve the planning of their actions, the monitoring of their understanding, the regulation of their emotions and motivation, robust beliefs of self-efficacy for learning, and a repertoire of individualized learning and study strategies that can be intentionally and proactively used to reach learning goals (Schunk & Greene, 2018; Weinstein & Acee, 2018; Zimmerman, 2013). However, strategic learning does not develop spontaneously. It needs to be taught, and teachers play a key role in this process. Thus, the present study investigates aspiring teachers in teacher education programs and provides original findings about the learning approaches used by these students and their beliefs about their capabilities to learn. Findings suggest a need for teacher education programs to not only teach students content and pedagogy, but also, and perhaps above all, to strengthen their self-regulated usage of learning strategies so that they can thrive in their teacher education program and be prepared to model and teach strategic learning to their future students. In fact, research suggests that teachers are far more capable of teaching their students how to learn, when they have developed knowledge and skills in how to learn effectively and efficiently (Boruchovitch & Ganda, 2013; Michalsky & Schechter, 2018; Moos & Ringdal, 2012).

## Limitations

Despite the contribution of the present study, it has some limitations that also need to be overcome by future research. One of them is related to the fact of the inventory of learning and study strategies for university students (Weinstein et al., 2016), translated and adapted by Boruchovitch et al. (2019), still being validated in Brazil. In general, the alfa values obtained in the present study were mostly acceptable and similar to those found in another Brazilian study (Boruchovitch et al., 2020). They were also somehow comparable to those obtained in American Studies (Weinstein et al., 2016), with the main exception of the using academic resource scale which was consistently very low in Brazilian samples. As the psychometric properties of the Brazilian version of LASSI are still under study, no items have been removed yet to increase reliability of this scale. However, this particular result is indeed intriguing and deserves further investigation in so far as it might reflect a cultural difference. Additionally, as part of the sample responded to the scales in the *Autorregular* Platform and part answered them in paper and pencil format, these differences in data collection procedures could have affected the results.

Moreover, data collection was based only on self-report measures and the sample size of the present study, by being composed of only one educational institution, with its own culture and teaching dynamics, does not allow the results to be seen as conclusive, nor able to be generalized.

## Future research

Considering the importance of understanding the impact of sociodemographic variables in the use of learning and study strategies and in self-efficacy for learning beliefs and the inconsistencies found in the literature regarding this impact, it is recommended that future research advance in this theme since this information has important implications not only for rethinking the curricula of teacher education programs, but also for designing interventions targeted at the improvement of the learning processes of university students as whole. Moreover, it is suggested that other variables such as period (full-time, morning, evening and night), type of high school students attended, ethnicity, academic performance, intention to continue the course, among others, be also examined.

It would be also interesting to exam the LASSI scales and the self-efficacy for learning beliefs scales in relation to other scales which investigate different psychological constructs associated with learning. Data emerging from these future studies will certainly provide a greater understanding of the different types of variables that are related to the learning process of future teachers. Further research should also include other measures than self-report which are more prone to bias.

It is also essential that further research be targeted at the teachers as well, since their role is to promote self-regulated learning and foster positive self-efficacy for learning beliefs in their students. They need to be involved and implicated in the discussion of findings from studies such as the present study so that they can tailor their actions towards empowering students to learn how to learn.

## Conclusion

Overall, results of the present study suggest that there is a need to teach strategies for students to regulate anxiety, since it was the scale that showed the lowest score. Investments should also be made regarding teaching strategies aimed at the utilization of academic resources, self-testing and time management. The present research also revealed that students in the sample reported having moderate beliefs of self-efficacy to learn. Sociodemographic variables were associated with strategic learning. Gender and licentiate area of students seem to be important for the understanding of academic anxiety, suggesting that female and students majoring in Biological Sciences, Chemistry, and Mathematics should undergo intervention that will help them better regulate their emotions. It was also identified the need to invest efforts to strengthen the learning and study strategies of students in the final semesters of their courses. It is hoped that these findings can contribute not only to improve the quality of teacher education programs and policies, especially in the areas of Natural Sciences and Mathematics, but also to increase research about self-regulated learning and teacher education, especially in a country in which investigation in this important area is still scarce.

## Data Availability

The raw data supporting the conclusions of this article will be made available by the authors, upon request, without undue reservation.
